# Non-realist cognitivism, superassertibility and vicious regress

**DOI:** 10.1007/s11229-026-05771-y

**Published:** 2026-08-19

**Authors:** Craig Patrick Ferrie

**Affiliations:** University of St Andrews and University of Stirling, St Andrews, Scotland, UK

**Keywords:** Meta-normativity, Truth pluralism, Superassertibility, Non-realist cognitivism, Epistemic iteration principles, Internalism/externalism

## Abstract

Normative truth is particularly mysterious for proponents of Derek Parfit’s ‘non-realist cognitivism’. On this view, for an irreducibly normative proposition to be true, irreducibly normative properties “need not exist either as natural properties in the spatio-temporal world, or in some non-spatio-temporal part of reality” (Parfit, 2011, p. 486). Parfit does not explain, however, how such propositions get to be true. One possibility builds upon Crispin Wright’s notion of “superassertibility” (1992, 1995). This view ties truth to the notion of indefeasible warrant, relative to an accessible state of information. Given that superassertibility is, itself, a normative notion, however, it might be thought that this suggestion entails some kind of vicious, infinite regress. This article will outline two distinct arguments, the first, courtesy of Jussi Suikkanen (2016) and the second, novel, that purportedly show that this leads to such a regress before demonstrating that, in light of some highly plausible, iterative principles of warrant, both arguments fail. Moreover, it will outline an entailment of these principles which shows that, in at least one important way, superassertibility is just like truth.

## Introduction

Intuitively, it is true that ‘you should not torture for fun’. Moreover, this proposition is, plausibly, about what you have *irreducibly* normative reason to do. This is to say that the reasons you have for not torturing (for fun) are not reducible to the satisfaction of your desires nor to facts about your engagement in some rule-governed activity, e.g., a game or etiquette. Moreover, an irreducibly normative reason is one that is not reducible to any natural facts, such as facts about pleasure or wellbeing (hereafter, ‘normative’ means irreducibly normative, unless otherwise specified).

Normative realists endorse normative truth but take this to position to be accompanied by ontological worries that need answered: what does normative existence consist of?^1^ Is this existence compatible with naturalism?^2^ How could we have epistemic access to normative existents?^3^ Normative anti-realists take these ontological worries to show that there is no normative ontology, and, as a result, often reject the possibility of normative truth.^4^

Derek Parfit (2011, 2017) contends that for a normative proposition to be true, normative properties “need not exist either as natural properties in the spatio-temporal world, or in some non-spatio-temporal part of reality” (2011, p. 486). According to Parfit then, normative truths have no ontological commitments. As such, he rejects the correspondence theory of truth, which says that a proposition is true if and only if it corresponds to reality (Parfit, 2017, p. 58). If this is the case then ontological worries need not undermine our intuitions that some normative propositions are true.

Parfit, however, fails to provide a positive suggestion for how one should think of normative truth. One possibility is that superassertibility plays the truth role for normative propositions. A proposition is superassertible if and only if (1) a state of accessible information warrants its assertion and (2) this warrant would survive “arbitrarily close scrutiny of its pedigree and arbitrarily extensive increments to or other forms of improvement of our information” (Wright, 1992, p. 48). This view grounds normative truth in the indefeasibility of some available warrant, or justification, for the target proposition, relative to an accessible state of information.

This article will present two distinct arguments that purport to show that this view leads to vicious regress. In Sect. 2, I will use Parfit’s view to springboard into the discussion, as the superassertibility theory of normative truth is, prima facie, particularly attractive for proponents of his view. In Sect. 3, I outline an objection, courtesy of Jussi Suikkanen (2016) which purportedly shows that the superassertibility theory entails vicious regress. In Sect. 4, I argue that Suikkanen assumes the falsity of an iterative principle of warrant (WW) which is intuitively correct. In Sect. 5, I respond to three possible objections which suggest that WW entails that warrant is unachievable. In Sect. 6, I argue that, despite appearances, WW is compatible with both internalism and externalism about warrant. In Sect. 7, I motivate a second, ‘stronger’ vicious regress objection, which is compatible with WW, before showing (in Sect. 8) that in light of a similar, iterative principle of superassertibility (SS), that it also fails. Finally, in Sect. 9, I highlight a welcome discovery that is unveiled by SS for all who think that superassertibility plays the truth role in some domain of discourse.

## Non-realist cognitivism

Parfit’s ‘non-realist cognitivism’ says the following,

There are some claims that are irreducibly normative in the reason-involving sense, and are in the strongest sense true. But these truths have no ontological implications. (Parfit, 2011, p. 484)^5^

Parfit endorses the ‘reason-involving’ conception of normativity. On this view a normative claim is one which “asserts or implies that we or others do or might have some reason or apparent reason” (2011, p. 268). Here, Parfit means that a normative claim is one which asserts or implies that we or others have a *normative* reason.^6^ Plausibly, then, a normative *proposition* is one which the assent of which asserts or implies that we or others do or might have some reason; assenting to the proposition ‘you should wear a helmet when cycling on the road’ then, is normative because it implies that you have reason to wear a helmet when cycling on the road. On this view then we can distinguish between normative propositions and descriptive propositions; the proposition ‘the door is brown’, for example, is not normative because it neither asserts, nor implies, that we, or others, have any kind of reason. Of course, one might think that ‘the door is brown’ implies the further proposition that ‘one should believe the door is brown’, but the former has no normative implication in and of itself. This view also explains why terms like ‘justification’, ‘warrant’ and ‘ought’ are normative terms: they are terms which, when asserted in a proposition, imply that we or others have some reason. The normative domain of discourse, then, is that which is united by those propositions that assert or imply normative reasons.

This view should be attractive for those who think that normativity is fundamentally the realm of reasons, like Scanlon (2014) and Parfit (2011, 2017).^7^ It need not be inconsistent, however, with those, like Broome (2018), who instead think that normativity is fundamentally about the ‘ought-relation’. One can think that a normative proposition is one the assent to which asserts or implies that we or others might have some reason whilst endorsing the view that what it means to have a reason is explained by appeal to the ought-relation.

Non-realist cognitivists reject the view that true normative propositions have ‘ontological implications’ and that such ‘reason-involving properties’ exist either as natural or non-natural entities within reality. This is, in part, because they reject what Parfit calls the ‘single sense view’ which says that ‘there are’ and ‘exist’ are always used in only one way (2011, p. 469). Instead, he adopts the ‘plural senses view’ which says that we can distinguish between a wide, general sense of the notions ‘there are’ and ‘exist’ and a ‘narrow actualist’ sense of them.

To say that x exists in the wide, general sense is not to claim that x is an object that can be found in reality; if x merely exists in the wide general sense then it is not a part of “metaphysical reality” (Parfit, 2011, p. 747). This means that wide, general existence claims do not carry ontological commitments to actual objects. In this sense we can say that reasons, numbers and fictional entities exist without there being any object in the world that these claims refer to. Moreover, it might be argued that this captures how we use such terms; it is thought, prima facie, that there are such things as reasons, numbers and fictional entities (we analyse and discuss them) but we do not, ordinarily, take this to entail a commitment to thinking that such things literally populate our reality in the same way that concrete objects do. The latter claim is a stronger (and far more controversial) claim than the former.

To say that x exists in the narrow, actualist sense, on the other hand, is to say that x really is an object that can be found in reality (at least in principle). Paradigmatic examples include the everyday concrete objects that we are all surrounded by every day, like cars, rocks and tables. Moreover, it is the narrow, actualist sense that is used when ecologists inform us that a species has gone extinct, e.g., there are no more thylacine, or when parents tell their children that Santa Claus does not exist. Of course, Santa exists in the wide, general sense but he fails to exist in the narrow, actualist sense because there is nothing in the world to which claims about Santa Claus correspond (he is not out there!). This is also a helpful way of framing the Platonists view of abstract objects; most of us are happy to contend that abstract objects exist in the wide sense, but Platonists make the stronger claim that they exist in the narrow sense (though not in space and time, unlike concrete objects).

Parfit thinks there is ‘no clear question’ as to what it could mean for abstract objects, like numbers and reasons, to exist in the narrow sense (2017, pp. 475–480). In what sense do they *actually* exist if they are not within space and time? As such, he thinks that it is unclear what it could mean to say that normative propositions are about objects in the world. Nonetheless, such claims, about numbers and reasons, seem capable of being true. And so, true normative propositions are not made true by their accurately describing, or corresponding to, reality.

Non-realist cognitivism can be contrasted with normative realism, which comes in two broad forms. A normative realist is either a naturalist who believes that normative properties are reducible to natural properties or a non-naturalist who believes that normative properties exist in the narrow, actualist sense, but outside of natural spatio-temporal reality, i.e., Platonism. Normative realists, of either form, get to say that some normative propositions are true in virtue of the fact that such propositions accurately represent the way reality is.^8^ The explanatory burden of naturalism is to explain which natural properties normative properties are reducible to. The burden of non-naturalism is to explain what it means for normative properties to exist, in the narrow, actualist sense, outside of space and time.

Non-realist cognitivists reject both naturalism and non-naturalism and so have neither explanatory burden. The cost, however, is that they do not get to say that a normative proposition is true if and only if it corresponds to some part of reality. This means that non-realist cognitivism is incompatible with a correspondence theory of truth.^9^ They also do not get to endorse a truthmaker theory of the kind endorsed by Mark Jago which says that “truth arises as a relation between a proposition—the content of our sayings, thoughts, beliefs, and so on—and an entity (or entities) in the world” (p. 1, 2018). If one thinks that normative propositions have no ontological implications and can nonetheless be true, then one rejects theories of truth, like the correspondence theory or truthmaker theory which take truth to be a relation between propositions and reality, or entities within the world.^10^ And so, the explanatory burden of the non-realist cognitivist is to explain how normative propositions get to be true without appealing to reality, or entities within it.

One possibility, not hitherto thoroughly investigated, is that Wright’s notion of superassertibility plays the truth role for normative propositions. Wright (1992, 1995) takes superassertibility to be a truth predicate given that it satisfies the minimal requirements of a truth predicate, i.e., it satisfies various truth platitudes. He motivates the view that superassertibility plays the truth role in mathematics and ethics. Other proponents include Edwards (2018), where superassertibility is said to play the truth role for a wide array of domains of discourse, and Lynch (2025), who argues that concordance plays the truth role in politics. Concordance differs from superassertibility in that it has, built into it, a coherentist theory of ‘warrant’ which simple superassertibility does not (Lynch, 2009). Moreover, ‘superassertibility’ is reasonably construed as a more technical version of the pragmatist view, originally proposed by Charles Peirce (1878), which says that a true belief is one that would withstand doubt, were we to inquire as far as we fruitfully could.^11^

Wright (1992) introduces the idea that superassertibility is a truth predicate for mathematical propositions as a way of explaining how anti-realists about mathematics can endorse mathematical truth. Anti-realism about a domain, thus understood, rejects the view that the objects of the domain (numbers, reasons, moral properties etc.) exist out there, in reality. In Parfitian terms, they reject the view that such objects exist in the narrow, actualist sense.^12^ Anti-realism then is a rejection of both naturalism and non-naturalism about a domain. Taking superassertibility to play the truth role in a domain is consistent with anti-realism about that domain because it makes no appeal to entities or properties in reality. Prima facie, then, the superassertibility theory of normative truth can be plugged into non-realist cognitivism; neither make any commitment to the existence of normative objects.

This proposal does not require one to endorse the view that the superassertibility theory be generalised across all truth-apt discourse. One might instead endorse alethic pluralism which allows us to endorse the view that superassertibility plays the truth role for normative discourse without committing us to the view that superassertibility plays the truth role for all discourse. For many, it is implausible, for instance, that superassertibility plays the truth role for natural propositions.^13^ Moreover, this need not require us to endorse ‘strong’ alethic pluralism which contends that there are a variety of truth properties.^14^ Instead, it is open to argue for *moderate* alethic pluralism which says that, although there are a variety of ways in which true propositions get to be true, that there is just one property of truth. Edwards provides a clear and compelling explanation of this view by way of analogy with the property of ‘winning’: just as there are a variety of ways that one might become a winner, depending on the game one is playing, it is plausible that there are a variety of ways for a proposition to become true, depending upon the subject matter of the proposition (2018, pp. 122–141).^15^

On the other hand, endorsing either form of pluralism is also not necessary for adopting the current proposal, as you could endorse the view that superassertibility plays the truth role for all domains of discourse. This is one way of interpreting the kind of Piercean pragmatism endorsed by the likes of Misak (2000).^16^ However, the proposal is, of course, incompatible with any theory of truth which says that any property, other than superassertibility (or some sufficiently similar property, like concordance) plays the truth role for all truth-apt discourse, e.g., the correspondence theory.

## Suikkanen’s regress

Suikkanen (2016) highlights the fact that the superassertibility theory sets a normative condition for truth; “a proposition is superassertible in part because information exists that would warrant asserting the proposition” and having warrant for an assertion “amounts to having justification and good reasons for doing so” (Suikkanen, 2016, p. 205). As Suikkanen points out, justification and reasons are normative notions. In considering how normative propositions get to be true then, if the answer is that they are true if and only if they are superassertible, then we must also explain the normativity that is “built right into the definition of superassertibility” (Suikkanen, 2016, p. 205).

This position, Suikkanen argues, leads the non-realist cognitivist into a trilemma. Firstly, they might adopt a different meta-normative view of epistemic normativity, i.e., about warrant. It is unclear what Suikkanen takes the upshot of this response to be, but presumably it would mean avoiding the superassertibility theory about epistemically normative propositions. The second option is to simply endorse a different theory of truth for epistemically normative propositions. Finally, they can endorse the superassertibility theory for all normative propositions. Suikkanen contends, however, that none of these options will work.

As Suikkanen highlights, adopting a different meta-normative view of epistemic normativity would be a very strange argument for the non-realist cognitivist to make as they would have to respond to their own objections to rival meta-ethical views (2016, p. 207).^17^ Parfit rejects non-naturalist realism on the grounds that it is simply not clear what it could mean for abstract objects, like normative reasons, to exist outside of space and time (2017, p. 60). Why should the existence of epistemic normative reasons be any clearer than the existence of practical normative reasons?^18^ Moreover, it is not clear what the upshot of this move would be, if not to allow the non-realist cognitivist to adopt a different theory of truth about epistemically normative propositions. This takes us on to the second horn of the trilemma.

Suikkanen’s proposed second option means adopting a different theory of truth about epistemically normative propositions. However, he provides independent reasons for thinking that non-realist cognitivism is incompatible with all ‘traditional’^19^ alternative theories of truth, including deflationism, pragmatism and coherence theory. As such, he concludes that the only possibility for the non-realist cognitivist who wishes to understand the truth of normative propositions by superassertibility, is to accept an unrestricted version of the view. And so, the non-realist cognitivist is unable to endorse a different theory of truth for epistemically normative propositions.^20^ As such, the non-realist cognitivist is committed to thinking that superassertibility propositions, like ‘P is superassertible’, also depend upon superassertibility for being true.

The third horn, which commits the non-realist cognitivist to endorsing the superassertibility theory of normative truth, simpliciter, purportedly leads to infinite vicious regress,

In order for the [normative] proposition to be superassertible and thus minimally true, it must also be true that some further information S* would warrant asserting that proposition. Of what does the truth of the proposition < INFORMATION S* WARRANTS ASSERTING THAT INFORMATION S WARRANTS ASSERTING THAT WE HAVE REASONS FOR AVOIDING FUTURE AGONY> then consist? If it consists of superassertibility too, then it must again be true that some further information S** warrants asserting that S* warrants asserting that S warrants asserting a proposition about our reasons. And, again, of what does the truth of this third-order epistemic normative claim consist? Here, the non-realist cognitivist would be committed to there being infinitely many states of information that would warrant asserting propositions about warranted assertion on the lower levels. It is not easy to see how there could be so many states of information. (Suikkanen, 2016, p. 206)

Suikkanen says then that if superassertibility plays the truth role for normative propositions, then for it to be the case that ‘P is superassertible’ is true, it must be the case that ‘P is superassertible’ is superassertible. He claims that for it to be the case that ‘P is superassertible’ is superassertible, there must be an informational state, S*, that warrants believing that ‘S warrants believing that P is warranted’. His argument says then that the warrant for believing that ‘it is warranted that P is warranted’ must be provided by a different body of information than S; S and S* are necessarily distinct.

This leads to the following regress,

1. P is true if and only if P is superassertible.
2. P is superassertible, in part, if information, S, warrants asserting that P.
3. ‘S warrants asserting that P’ is true if and only if ‘S warrants asserting that P’ is superassertible.
4. ‘S warrants asserting that P’ is superassertible, in part, if information, S*, warrants asserting that ‘S warrants asserting that P’.
5. ‘S warrants asserting that S warrants asserting that P’ is true if and only if ‘S warrants asserting that S warrants asserting that P’ is superassertible.
6. ‘S warrants asserting that S warrants asserting that P’ is superassertible, in part, if information, S* warrants asserting that ‘S* warrants asserting that S warrants asserting that P’.
  -ad infinitum

Suikkanen takes this regress to be vicious because in order to endorse the superassertibility theory, the non-realist cognitivist is committed to there being “infinitely many states of information that would warrant asserting propositions about warranted assertion on the lower levels” (2016, p. 206). There are infinitely many states of information because Suikkanen assumes that it could not be true that S and S* (and S**, etc.) are the same body of information. He says that S would have to be,

(i) accessible to ordinary inquirers and (ii) independent of human judgements and attitudes and (iii) the information that warrants a given normative statement would also have to be conceptually independent of the statement that is being warranted. (Suikkanen, 2016, p. 206, fn307)

It is not clear why he assumes that the information states would have to be conceptually independent of one another. Nonetheless, he claims that these conditions “rule out comprehensive information states and the original statement itself from playing the required warranting role” (2016, p. 206).^21^

This infinite series of states of information is taken to be vicious because it is “not easy to see how there could be so many states of information” (Suikkanen, 2016, p. 206). Suikkanen’s worry here is that there cannot be infinitely many states of information that play the warranting role in believing propositions. In order for this to be the case, not only would it need to be true that there exist infinitely many states of information (which seems correct!), but that we have access to such an infinity of states of information. It is part of the notion of superassertibility that it is grounded in accessible, warranting information. The regress seems to suggest, however, that we would need access to an infinite series of states of information for it to be the case that some proposition is superassertible. This, Suikkanen suggests, is not possible.

## The principle of warrant (for warrant)

For Suikkanen’s regress to generate it must be a possibility that the lower-order proposition, ‘P is superassertible’ obtains whilst the higher-order proposition, ‘P is superassertible, is superassertible’ does not. Were this not a possibility then there would be no regress: the obtaining of the lower-order proposition would entail the obtaining of the higher-order and thus, where P is superassertible, so too is the subsequent higher-order proposition. This would also entail that all subsequent higher-order propositions also obtain.

More specifically, it must be possible that one is warranted in believing P but not warranted in believing that ‘P is warranted’. If it is not a possibility that the lower-order proposition obtains but the higher-order proposition does not obtain, then the regress collapses. Suikkanen’s regress therefore depends on the assumption that there can be sufficient warrant for believing P even if there is not sufficient warrant for believing ‘P is warranted’. So, the question is whether this a plausible assumption to make, can it be the case that P is warranted even if ‘P is warranted’ is not warranted?

It is difficult to imagine how this could be the case. Imagine the weather person declares that, given the current weather patterns, the evidence permits believing that tomorrow it will rain in Scotland and so, we are warranted in believing that ‘tomorrow it will rain in Scotland’. Now imagine the weather person declares that this belief, ‘tomorrow it will rain in Scotland is warranted’ is not itself warranted. So, although we (epistemically) ought to believe (or it is permissible to believe) that ‘tomorrow it will rain in Scotland’, it is not the case that we ought to believe (or it is permissible to believe) that ‘tomorrow it will rain in Scotland is warranted’. Presumably, this would lead to confusion: how could it be the case that we are warranted in believing that it will rain if we are not warranted in believing that we are warranted in believing it will rain. If we do not have warrant for the claim that ‘tomorrow it will rain in Scotland is warranted’ then this would seem to entail that we do not have warrant for the claim that ‘tomorrow it will rain in Scotland’. And so, the weather person’s declaration seems to be incoherent.

Moreover, it is difficult to understand why the weather person would want to make this claim. If they believe that ‘tomorrow it will rain in Scotland’ is warranted then for what reason would they doubt that ‘tomorrow it will rain in Scotland is warranted’ is warranted? It seems that whatever reason the weather person has for declaring that ‘it will rain in Scotland’ is warranted also provides reason for declaring that ‘it will rain in Scotland is warranted’ is warranted.

Another way of putting this is to say that, if there is reason to doubt the higher-order claim then it seems we have just as much reason to doubt the lower-order claim. If the warrant for the higher-order warrant is defeated, and so we shouldn’t believe that we have warrant to believe ‘tomorrow it will rain in Scotland is warranted’, then surely, we should not believe that we have warrant for the lower-order claim either. It would be awfully strange to claim that there is sufficient reason to doubt that ‘P is warranted’ is warranted and claim that, nonetheless, there is not sufficient reason to doubt that P is warranted. If there is reason to doubt that ‘P is warranted’ is warranted then this seems to entail that there is reason to doubt that P is warranted.

Put simply, we assume that without higher-order warrant there can be no lower-order warrant for a proposition. As such, it seems to be the case that if P is warranted, then ‘P is warranted’ is also warranted. If this is correct, then Suikkanen’s regress is not generated. If warrant entails warrant for warrant then there is no need to appeal to some further body of accessible information in motivating the claim that ‘P is warranted’ is warranted. Suikkanen therefore needs the following iterative principle to be false,

(WW) if P is warranted then ‘P is warranted’ is warranted.

If the warrant for P entails warrant for all subsequent propositions, then there is no vicious regress; if one is warranted in believing P then one is also warranted in believing P*, P**, P*** etc.

If WW is correct then, as alluded to earlier, the state of information, S, that warrants P, is the same body of information that warrants P*, P**, P*** etc. This is because, for P to be warranted, P*, P**, P***, etc. must also be warranted. The state of information that warrants P then, must be the same state of information that warrants P*, P**, P***, etc. So, if S warrants believing P then S also warrants believing all subsequent, higher-order superassertibility propositions.^22^^,^^23^

## Does WW render warrant unachievable?

It might be objected that WW makes warrant too difficult to ever achieve. You might think, for instance, that each higher-order claim (P*, P**, P***, etc.) is a stronger claim than the last. On the face of it, it takes more for ‘P is warranted, is warranted’ to be true than it does for ‘P is warranted’ to be true as the former claim is defeated in more conditions than the latter. ‘P is warranted’ is false just in case (1) there is not a state of information that warrants believing P, whereas ‘P is warranted, is warranted’ is false in case, either (1) there is not a state of information that warrants believing P or (2) there is not a state of information that warrants believing ‘P is warranted’. If this is correct, and if S must play the role of warranting not only P but every other higher-order proposition in the regress, then it is just not plausible that P will ever be warranted. This is because, presumably, there is no state of information that warrants an infinitely long list of increasingly stronger propositions. As such, P can never be warranted.

WW seems to show, however, that this is not the case. If the truth conditions for ‘P is warranted’ and ‘P is warranted, is warranted’ are the same, i.e. whenever ‘P is warranted’ is true “P is warranted, is warranted’ is true, then the latter cannot be a stronger claim than the former. If there are more possible worlds in which the latter is false than the former then the truth conditions are not the same. So, if WW is true, then the higher-order claim is not a stronger claim, for which more is required to obtain, for it to be true. And so, WW does not entail that warrant is unachievable. Instead, it entails that ‘P is warranted, is warranted’ is not a stronger claim than ‘P is warranted.^24^

This might seem strange, but the suggestion is not without precedent as this is how truth operates: if ‘P is true’ then it follows that ‘P is true, is true’, and vice versa. And it clearly seems that the latter, higher-order claim, is not any stronger than the former; nothing more is required for the latter claim to be true than is required for the former claim to be true. In the case of truth, this does not seem strange at all. So, the suggestion is that warrant, is just like truth in this way. And so, the idea that the higher-order warrant claims, P*, P**, etc. are not any stronger than the lower-order claims, is not unique to warrant.^25^

Another sceptical worry arises for iterative principles which require that if you believe P, then you believe that you believe P. For example, KK says that if you know P then you know that you know P (Williamson, 2000). Let’s assume that a necessary condition of knowledge is belief – that is, it is necessarily the case that if you know P, then you believe P. Given KK, this means that if you know P then, necessarily, you also believe that you know P. These beliefs iterate infinitely, and so, it must be the case that if you know P then you have an infinitely long list of iterating beliefs about P, i.e., that you believe that you know, that you know, that you know, that you know P (ad infinitum). The first worry is that we, of course, don’t appear to have these beliefs: in believing P one does not, typically, have an infinitely long list of iterative beliefs about P. And secondly, it is not clear that it is even possible that we could have such an infinitely long list of beliefs. If so, then iterative principles which entail that we do, or could, believe such an infinitely long list of beliefs, should be rejected.

Moreover, it might be objected that even if it is the case that we can have an infinitely long list of beliefs that, at some stage in the regress, the propositions become too complex to be believed. It might be thought, for instance, that it is not possible to believe ‘it is known, that it is known, that it is known, that it is known, that P is known’, because it is too complex. If this is the case, then, similarly, it can never be the case that one knows that P. So, alongside the worry that an iterative principle might entail beliefs too long to hold in one’s mind, there is the worry that such a principle might entail beliefs too complex to understand. 26

Whether these objections can be levelled at WW depends on whether WW is a principle of propositional or doxastic warrant. For P to be propositionally warranted it is simply the case that there is a state of information which warrants P being believed (and so it does not need to be actually believed). Doxastic warrant, on the other hand, demands that (i) P is warranted, relative to a state of information and (ii) P is believed on the basis of the information that warrants the proposition (Feldman, 2002; Kvanvig, 2003). As such, propositional warrant is a more basic notion than doxastic warrant; doxastic warrant is just propositional warrant with the added condition that P is actually believed, on the basis of the relevant information.

WW, however, only requires the condition that P is warranted, relative to a state of information. As such, there is no reason to construe it doxastically: it does not demand that P is actually believed. This is consistent with Suikkanen’s regress because it is also best understood in terms of propositional warrant: it makes no mention of any of the relevant propositions actually being believed. And this is because superassertibility is best understood in terms of propositional warrant: it is not the case that for P to be superassertible that P is actually believed. And so, there is no reason to pursue a doxastic WW principle. Suikkanen’s regress and WW are both talking about propositional warrant.

Moreover, such a principle should likely be rejected: it is highly plausible that one believes P and is (propositionally) warranted in believing P, even if they do not believe ‘P is warranted’. This means that one can be doxastically warranted in believing P even if they are not doxastically warranted in believing ‘P is warranted’ because they fail to meet the second condition of doxastic warrant (as they do not actually believe ‘P is warranted’). This suggests that doxastic warrant does not iterate and we should, therefore, reject a doxastic variant of WW.

This means that it is not the case that WW requires that if P is warranted then one actually believes every proposition in the regress (because warrant does not require belief). And so, neither objection can be levelled at WW: warrant for P does not demand that one actually believes an infinitely long list of propositions nor that one believes one of the incredibly complex propositions within the regress.

It might nonetheless be objected that even if propositional warrant does not require actual belief, it does entail *possible* belief (hereafter, ‘warrant’ means propositional warrant). This is to say that if P is warranted then it must be possible to believe P. And so, if the infinite list is not possible to believe, or the propositions become too complex to be believed, this still undermines WW. This is because WW entails that if P is warranted then every proposition in the regress is also warranted. If warrant entails possible belief then this means it must be possible to believe both the infinite regress and every (complex) proposition within the regress. Assuming these are not possible to believe, this entails either that WW is false and warrant for P does not entail warrant for ‘P is warranted’ or that WW is correct and warrant is unachievable.

However, comparison to some highly plausible logical principles shows that this is likely not the case. It is highly plausible, for instance, that warrant is closed under conjunction: if P is warranted and Q is warranted then ‘P and Q’ is also warranted.^27^ From this we can infer that the conjunction of every warranted proposition is also warranted. It is similarly unclear, however, how such a long conjunction could ever be believed. Nonetheless, it is surely warranted. And the same can be said for disjunction: if the proposition, P, is warranted, then any disjunction involving P, as a disjunct, is also warranted. A disjunction is true if at least one of its disjuncts are true, and so, if at least one of its disjuncts are warranted then the entire disjunction is warranted. But this is correct even when we recognise the possibility of constructing a disjunction so long – even infinite - that seems too complex to be believed. If we want to maintain these very plausible principles, we must either think that these propositions can be warranted even though it is not actually possible to believe them, or that they are somehow in fact believable. Either way, the problem is avoided.

Greco (2015), for instance, thinks that it may be plausible that we can hold the kinds of infinitely long and complex beliefs that are objected to. The intuitive pull of the objection to infinite beliefs, he suggests, depends upon our accepting an outdated “crude sentence-storage” (2015, p. 767) picture of belief which says that if one believes P then one has a “sentence-sized representation” (2015, p. 767) of P in one’s mind. If this is the case then it is difficult to understand how we could have the mental space for an infinite series of beliefs. He thinks, however, that this view of belief is false: there are kinds of beliefs one can have that are not constituted by mental representations in one’s mind (dispositional beliefs, perhaps). He also thinks that it need not be the case that complexity rules out belief. It is likely the case, for instance, that you believe that there are not any demons waiting for you to get home. And so, you likely believe that there are not exactly two demons, and you believe that there are not exactly three demons, etc. It is plausible, however, that natural numbers “increase in complexity without bound” (2015, p. 767). And so, each new belief in the series increases in complexity, without bound. Given that it is plausible that you could believe each member of this series, it is not evident that there is a “distinctive difficulty involved in holding highly complex beliefs” (2015, p. 767). As such, it is not evident that infinitely long, and highly complex beliefs, are beyond our believing.^28^

## Does WW require internalism?

It might be thought that endorsing WW requires endorsing an internalist theory of warrant. Internalists endorse some form of accessibility condition on warrant: if one is warranted in believing P then, necessarily, they can, by reflection (introspection, a priori reasoning, memory etc.), access the information that warrants P (Kelp and Pederson, 2013). Externalists, on the other hand, deny this claim. For the externalist, you might have warrant to believe a proposition even if you can’t access the information that warrants P. If it were the case that WW required internalism this would mean that non-realist cognitivists who defend the superassertibility theory are also committed to endorsing internalism. This would limit the scope of the argument against Suikkanen’s regress and could be framed, by externalists, as a reason to reject it. 29

Moreover, internalism seems like a natural fit for the superassertibility theory. If P is superassertible then the warrant for P is necessarily epistemically accessible. This sounds a lot like internalism; if P is warranted then it is possible to access the information that warrants it’s being believed. So, you might think that internalism is the natural view of the proponent of the superassertibility theory.

So why think that WW requires internalism? Well, Smithies (2019) endorses a similar iterative principle about justification which is explicitly internalist in character. The JJ principle says that “[n]ecessarily, you have justification to believe that p if and only if you have higher-order justification to believe that you have justification to believe that p” (2019, p. 261). Smithies takes ‘higher-order justification’ to be justification that necessarily comes from reflection, “[i]f you reflect in a way that is fully justified, then you will form the justified higher-order belief that you have justification to believe that p, and you’ll thereby come to believe that p” (2019, p. 263). As such, Smithies’ JJ principle understands higher-order justification to be necessarily internalistic. If WW is analogous to JJ then it is also internalistic.

WW, however, is broader than JJ and does not commit one to an internalist account of warrant. WW, unlike JJ, only makes use of one notion of ‘warrant’ (use of ‘higher-order’ is used in the discussion for clarity and is eliminable). Strictly speaking, the principle does not rely on ‘higher-order warrant’ and so, the meaning of ‘warrant’, whether ‘higher-order’ or ‘lower-order’, should be understood as staying consistent. As such, if one endorses internalism then both the warrant for P and the warrant for ‘P is warranted’ should be construed internalistically or if one endorses externalism then both should be construed externalistically. As such, it does not require that when we move from warrant for P to warrant for ‘P is warranted’ that we move toward an internalistic notion of warrant, as JJ does. It says nothing about accessing, by reflection, the warrant one has for believing P or for believing ‘P is warranted’. And so, there is no reason to think that WW, itself, commits one to internalism.

Moreover, note that in Wright’s initial formulation of superassertibility, it is not assumed that ‘warrant’ is epistemically available to us by reflection. Instead, it is stipulated that the relevant information states that provide the warrant are necessarily epistemically accessible, and the proposition attains the status if the warrant would survive all forms of improvement of our information. So, the epistemic accessibility of superassertible propositions comes not from the fact that the relevant notion of warrant is an internalistic one, but from the relevant kinds of information: accessible information.

It might be responded that, without motivating internalism about warrant, assuming the relevant information is epistemically accessible is unmotivated: why think that the warrant arises from accessible information if one does not already think that what is warranted is accessible to us. But this claim can be motivated without appealing to internalism. For instance, if one believes that all information within some domain of discourse is necessarily epistemically available to us. Wright himself argued that mathematical truths cannot escape proof (1992, 1995), Lynch (2025) thinks that political truths must be capable of being known, various kinds of ‘social’ truths must be necessarily knowable (Edwards, 2018), and many have thought, for different reasons, that moral, or normative truths, should be epistemically accessible to us. It is natural to think, for instance, that normative truths imply praise and blame and that normative claims are action-guiding claims. Neither of these conditions make much sense if it is not possible to know the relevant normative truths.^30^ As such, it is possible to motivate the claim that the relevant information is necessarily epistemically accessible to us without assuming internalism about warrant.

It might be argued that, even if WW does not strictly require endorsing internalism that externalists will not find WW plausible. According to reliabilism (one form of externalism), whether one’s belief is warranted can depend on whether it was produced by a reliable belief-formation mechanism (relative to one’s circumstances). As one reviewer suggested, however, it seems both possible and intuitive that one’s belief that P (that’s a barn) can have been produced by a reliable mechanism and hence be warranted, when you are not in fake-barn country, even if your belief that your belief is warranted is not warranted, as you lack a reliable mechanism to track which of your belief-formation mechanisms are reliable (in one’s current circumstances). If this is what WW demands, on an externalist account of warrant, then the externalist will not find WW plausible.

Whilst this is intuitive and possible, this response again moves from an externalist account of warrant, when assessing the warrant of P relative to a reliability condition, to an internalist account of warrant, when assessing the warrant of the belief ‘P is warranted’ relative to the *tracking* of one’s belief-formation mechanisms. Presumably, this tracking requirement just is the access requirement that internalists endorse; if one can track their reliable belief-formation mechanism than one can, by reflection, access the information that warrants believing P. As such, WW does not make this demand on an externalist account of warrant. Instead, the externalist can endorse externalism about warrant all the way up.

Furthermore, once the tracking condition is removed, the principle is much more plausible. It is plausible that the same reliable mechanism that warrants believing P (that’s a barn) also warrants believing that ‘P is warranted’. If you are warranted in believing that’s a barn, given the reliability of your recognising barns through your sense perception, then this same mechanism warrants believing that your beliefs which are warranted relative to your sense perception are also warranted. Presumably, if you have warrant to believe P because your sense perception is reliable then you also have warrant to believe that ‘you have warrant to believe P’.^31^

## A stronger regress

It has been argued that ‘P is warranted, is warranted’ is not a stronger claim than ‘P is warranted’. Nonetheless, to say that ‘P is superassertible’ is a stronger claim to make than to say that ‘P is warranted’. For P to be warranted it only needs (1) to be warranted, relative to a state of information. To be superassertible, on the other hand, P needs (1) to be warranted, relative to a state of information, and (2) that the warrant for P remains despite thorough investigation and all improvement of that state of information. Included in the proposition that P is superassertible then is a condition of indefeasibility; P is superassertible when P is indefeasibly warranted relative to some state of information. As such, there are conditions such that ‘P is warranted’ is true but ‘P is superassertible’ is not true, e.g., when the warrant for P is not indefeasible. In this way, ‘P is superassertible’ is a stronger proposition than ‘P is warranted’.

Similarly, it might be thought that the proposition, P**, ‘it is superassertible that P is superassertible’ is also a stronger proposition than, P*, ‘P is superassertible’. This is because P** appears to be defeated in more conditions than P*. P** is false if any of the following four conditions are not met; (1) P is warranted, relative to a state of information, (2) the warrant for P remains despite thorough investigation and all improvement of that state of information, (3) P* is warranted, relative to a state of information, and (4) the warrant for P* remains despite thorough investigation and all improvement of that state of information. If this is correct, then P** has four defeaters whilst P* only has two. Therefore, P** is a stronger proposition than P*. By extension, this suggests that, P***, ‘it is superassertible that it is superassertible that P is superassertible’ is a stronger proposition than P**. P*** appears to have six defeaters, whilst P** only has four.

If this is correct then it might be thought that we can develop the following regress worry,

1. P is true if and only if P is superassertible.
2. ‘P is superassertible’ is true if and only if ‘P is superassertible’ is superassertible.
3. ‘P is superassertible is superassertible’ is true if and only if ‘P is superassertible is superassertible’ is superassertible.
  -ad infinitum

This regress seems to entail that there are some propositions that we could never be warranted in believing despite their supposedly being superassertible. The further down the regress one goes, the more is required for each proposition to be true (as there is more required for it to be superassertible), and so more is required for one to be warranted in believing each new proposition. If this goes on forever, however, there presumably comes a point in which one cannot be warranted in believing such a proposition. This undermines the view that superassertibility plays the truth role for normative propositions because, it entails that there are propositions that are superassertible of which one cannot have warrant to believe. This should not be possible.

Moreover, this regress cannot be resolved by WW; it says nothing about needing warrant for a proposition about warrant. This regress also does not depend upon the assumption that the warrant for P* necessarily comes from a different body of information than the warrant for P. As such, neither the adoption of WW nor the denial that the warrant for P and P* come from different bodies of information will undermine the regress.^32^ This regress problem is therefore not susceptible to the objections that undermine Suikkanen’s regress.

## The principle of superassertibility (for superassertibility)

If a proposition is stronger than another then, necessarily, it is a possibility that the stronger proposition is false, whilst the weaker proposition is true. This is because if all of the conditions are met for the stronger proposition then all of the conditions are met for the weaker proposition, but not vice versa. As such, it is a possibility that the stronger proposition is false whilst the weaker proposition is true. In order for it to be the case then that the proposition ‘it is superassertible that P is superassertible’ is a stronger proposition than ‘P is superassertible’, it must be possible that the weaker proposition is true whilst the stronger proposition is false.

If WW is true, however, this is not possible. To say that ‘P is superassertible’ is, in part, to say that ‘P is indefeasibly warranted’ (relative to a state of information). So, to say that ‘P is superassertible, is superassertible’, is, partly, to say that ‘P is indefeasibly warranted, is indefeasibly warranted’. But how could it be the case that ‘P is indefeasibly warranted’ if ‘P is indefeasibly warranted’ is not itself indefeasibly warranted. If ‘P is indefeasibly warranted’ is not indefeasibly warranted this means that it will be defeated at some stage in an inquiry. But if this is the case, and WW is true, then the defeat of ‘P is indefeasibly warranted, is indefeasibly warranted’ entails the defeat of ‘P is indefeasibly warranted’. This is because if ‘P is indefeasibly warranted, is indefeasibly warranted’ is defeated, then ‘P is indefeasibly warranted’ is no longer warranted and this entails that P is no longer warranted. If P is not warranted then it is not indefeasibly warranted. And so, it is not possible that P is indefeasibly warranted if ‘P is indefeasibly warranted’ is not indefeasibly warranted. This means that where there is indefeasible warrant for P there is, necessarily, indefeasible warrant for ‘P is indefeasibly warranted’. This motivates the following principle,

(IW) if P is indefeasibly warranted then ‘P is indefeasibly warranted’ is indefeasibly warranted.

If WW and IW are true then it follows that if P is superassertible, then ‘P is superassertible’ is superassertible. This is because, according to WW, condition (1) of superassertibility, of warrant, relative to a state of information, entails warrant for that warranted proposition, relative to that state of information, and, according to IW, condition (2), that the warrant is indefeasible, relative to a state of information, entails indefeasible warrant for that indefeasibly warranted proposition, relative to a state of information. As such, both conditions required for a proposition to be superassertible operate in the same way. From this we can extrapolate the following principle of superassertibility,

(SS) if P is superassertible then ‘P is superassertible’ is superassertible.^33^

This entails that the propositions in the stronger regress do not grow in strength. If a proposition is stronger than another proposition only if it is possible for the weaker proposition to be true whilst the stronger proposition is false then SS says that ‘P is superassertible, is superassertible’ is not a stronger proposition than ‘P is superassertible’. This is because it is not possible for ‘P is superassertible, is superassertible’ to be false whilst ‘P is superassertible’ is true.

As such, it is false that there are more conditions in which ‘P is superassertible’ is superassertible’ is false than ‘P is superassertible’. If it is not a possibility that the former proposition is false whilst the latter is true, then it follows that where the former is false the latter is false, and where the former is true, the latter must also be true. Both propositions have the same truth conditions. The proposition ‘‘P is superassertible’ is superassertible’ then, does not have more defeaters than ‘P is superassertible’. And so, despite appearances, the higher-order proposition is not stronger than the lower-order proposition.

If SS is true, then there is no epistemic regress; there is no hierarchy of strength. If there is indefeasible warrant for believing P then there is indefeasible warrant for believing P*, P**, P*** etc. And so, if P is superassertible then so too are all higher-order propositions in the chain.

The objections levelled at WW in Sect. 5 might similarly be levelled at SS. Given that SS entails that higher-order superassertibility propositions do not increase in strength, however, this undermines the first objection which would say that SS entails that superassertibility is unachievable. If it were the case that the propositions increased in strength, and that ‘P is superassertible’ obtains only if ‘P is superassertible, is superassertible’ obtains (and this goes on infinitely) then it is difficult to see how an information state, S, could ever provide the indefeasible warrant for P. But, as is the case with WW, SS entails that these propositions do not grow in strength and so it does not undermine the possibility that S provides indefeasible warrant for P.

Moreover, the discussion in Sect. 5 on the implausibility of holding infinite beliefs, or complex beliefs, appears sufficient to undermine any of these objections to SS. Superassertibility does not entail actual belief, as it is depends on propositional, rather than doxastic, warrant, and so there is no reason to think that in order for P to be superassertible one needs to actually believe the infinity of (complex) beliefs. And it is not evident that SS should be rejected on the grounds that superassertibility entails possible belief. Assuming that superassertibility, like warrant, is closed under conjunction and that any disjunction where one of its disjuncts is superassertible, is also superassertible, then the argument from Sect. 5 follows. Accepting these highly plausible principles entails believing either that superassertibility does not entail possible belief, or that infinite and worryingly complex beliefs actually can be believed. As outlined in Sect. 5, Greco (2015) motivates the latter claim. In either case, the objection is diffused. 34

Finally, given the dependence, and similarity in form of SS and IW to WW, there is no reason to think that SS and IW require internalism about warrant: both SS and IW are compatible with both internalism and externalism about warrant (as discussed in Sect. 6).

## The principle of truth (for truth)

SS entails an added, positive entailment of my argument; it shows that in at least one vital sense superassertibility behaves just like truth. If P is true, then it is true that P is true; this is a tautology. It therefore follows that, if P is true then it is also true that it is true that P is true, ad infinitum. There is no viciously epistemic regress facing truth because this regress does not grow in strength. If the truth of P entails the truth of ‘P is true’, then knowing that P is true is sufficient for knowing that ‘P is true’ is true (and so on). As such, there is no epistemic mystery facing ‘truth’ and we should accept an iterative principle of truth,

(TT) if P is true then ‘P is true’ is true.

As outlined in the previous section, ‘superassertibility’ behaves in precisely the same way. According to SS,

(SS) if P is superassertible then ‘P is superassertible’ is superassertible.

Superassertibility then, aptly, behaves in the same way as truth; there is no epistemic regress of strength facing the superassertibility theory of normative truth. Were this not the case it would, prima facie, constitute a reason for rejecting superassertibility as a truth property. If TT is correct but SS is not correct, then it is unclear whether it could be possible that P is true if and only if it is superassertible. This is because, if P is true, then it necessarily follows that ‘P is true’ is true. If P is true if and only if P is superassertible however, it seems it would necessarily have to follow that if P is superassertible, then it is superassertible that P is superassertible. If this were false, then it is unclear how it could be the case that superassertibility plays the truth role in any domain of discourse. If superassertibility plays the truth role in normative discourse then it ought to behave in the same way that truth itself behaves; if truth entails truth for truth then superassertibility ought to entail superassertibility for superassertibility.

That truth and superassertibility behave similarly in this way undermines one possible objection to the view that a normative proposition is true if and only if it is superassertible. Were it the case that increasingly regressive superassertibility propositions also increased in strength then this would mean that superassertibility behaves differently from truth in one important respect. That truth and superassertibility do not differ in this way is a significant discovery for all who think that superassertibility plays the truth role in some domain of discourse.

## Conclusion

In summary, it looks like there is no vicious regress facing the superassertibility theory of normative truth. Endorsing either of the worries outlined in this paper requires rejecting highly plausible principles of warrant. It has also been shown that these principles do not entail that warrant is unachievable, nor do they require endorsing internalism about warrant. Moreover, these principles unveil a welcome feature of superassertibility for those interested in taking it to be a truth predicate in any domain of discourse. As such, the view that a normative proposition is true if and only if it is superassertible looks promising, particularly if you are a non-realist cognitivist.

## Data Availability

Not applicable.

## Footnotes

1 See Parfit’s ‘no clear question’ objection (2017, pp. 475–480).

2 Olson (2014, pp. 116–138) is plausibly interpreted as arguing that it is because normative reasons are not compatible with metaphysical naturalism that they do not exist.

3 Shafer–Landau (2003) provides a realist defence against these ontological worries and Enoch (2011) provides a defence of ‘Robust Realism’.

4 Mackie (1977) and Olson (2014), for instance, explicitly reject (some) normative truth for these reasons.

5 Parfit (2011) calls this view ‘non-metaphysical cognitivism’. It has recently been defended by the likes of Akhlaghi (2022), Karimi (2024), Jack (2025) and Jack and Khuramy (2025).

6 Such a reason is one that counts in favour of some action, belief or emotion. This contrasts with explanatory reasons which merely outline a causal relation between some phenomenon and its cause, or a motivating reason which refers to the psychology of an agent.

7 At least when it comes to reasons for action, in contrast to reasons for belief or feeling.

8 Jack and Khuramy (2025) usefully explain these views in terms of truthmakers. The non-naturalist thinks that normative propositions have abstract objects, outside of space-and time, as their truthmakers and the naturalist thinks that normative propositions have concrete, natural and spatio-temporal objects as their truthmakers. A truthmaker is something such that its existence makes some corresponding proposition true. They instead then layout non-realist cognitivism as the view that claims that there are truthmaker gaps, in the case of true normative propositions. This is to say that for normative propositions, there are truths without truthmakers.

9 Moreover, Parfit explicitly rejects correspondence theory (2017, p. 58). Suikkanen (2016) and Jack and Khuramy (2025) also argue that non-realist cognitivism is incompatible with correspondence theory.

10 This also suggests that either correspondence theory or truthmaker theory is what realists and error theorists each tacitly endorse.

11 See Misak (2000) for a contemporary defence and application of Peirce’s pragmatism.

12 As such, I take ‘anti-realism’ to be synonymous with Parfit’s ‘non-realism’.

13 Lynch’s ‘scope problem’ (2009) uses this consideration to motivate alethic pluralism. Dodd (2012) argues that it does not.

14 Edwards provides a discussion of compelling arguments against this view (2018, pp. 122–141).

15 Edwards’ (2018) own version of the view says that there a variety of properties that determine truth (2018). Lynch, similarly, thinks that there are a variety of properties that the single property of truth is manifested by (2009). See, Pederson and Wright (2012) for more discussion on the debate between strong and moderate alethic pluralism.

16 Though she herself rejects this interpretation (2000, pp. 70–85).

17 As an anonymous reviewer mentioned, it is a possibility that (some kind of) naturalism about epistemic normativity avoids Parfit’s objections to rival meta-ethical views, and so this might be one option the non-realist cognitivist could take. Alternatively, one could take the view that epistemic normativity is not robustly normative, i.e., reasons for belief do not have the normative force that irreducible reasons for action do. If either is correct then, contra Suikkanen, the non-realist cognitivist does have the option of adopting a different meta-normative view of epistemic normativity.

18 Jack and Khuramy (2025) argue that the non-realist cognitivist could adopt a different normative view about epistemic normativity, but as their paper only aims to outline possible routes the non-realist cognitivist could entertain, they fail to provide independent motivation for such a move. As such, it is not clear how such a move could avoid charges of ad-hocness.

19 ‘Traditional’ is used to exclude primitivism about truth which Suikkanen argues is the only compatible theory of truth for the non-realist cognitivist. He goes on to argue however, that the non-realist cognitivist ought to reject primitivism for independent reasons.

20 Jack and Khuramy (2025) argue that Suikkanen actually fails to show that non-realist cognitivism is incompatible with deflationism, pragmatism, coherence theory and correspondence theory. Though I think much of what they argue is likely correct, it is beyond the scope of this paper to work through their arguments, so I will instead accept Suikkanen’s arguments at face value.

21 Jack and Khuramy (2025) challenge this claim but do not explain why Suikkanen is wrong to make it. In Sect. 4, I show why Suikkanen’s claim should be rejected.

22 And as such, this argument provides motivation for Jack and Khuramy (2025) who argue that the information states need not be distinct.

23 Alternatively, it might be argued that although S* is distinct from S that S* could be a subset of S. If this is true then when S is true S* is also necessarily true. This would mean that S and S* are distinct but that it is not possible that ‘P is warranted’ is true and ‘it is warranted that P is warranted’ is false. If this is the case, however, the regress is not generated; having access to S provides access to S*, S**, S*** etc. As such, Suikkanen’s regress is incompatible with the view that S* is a subset of S.

24 This likely pre-empts any concern that Williamson’s ‘margin for error’ objection (2000) could be levelled at WW. The objection, typically levelled at KK, assumes that if one knows that P then they couldn’t easily have been wrong that P. It is then assumed that knowing that one knows that P is more demanding than knowing that P, and so knowing that you know that P could be more easily wrong than simply knowing that P. As such, KK is false; one can know that P without knowing that they know. The analogue of this, levelled at WW, says that it could more easily be the case that you are not warranted in believing you are warranted in believing P. But, if the higher-order claim is not stronger than the lower-order claim then there is no reason to think this is so. Moreover, it is not at all clear why we should accept that one is warranted in believing P only if they could not easily be wrong that P, as is required for the objection.

25 Warrant in this way resembles the infinite iterability of truth, which will be returned to in Sect. 9.

26 See Fumerton (1995, p. 64) and Bergmann (2006, p. 15) for these kinds of worry.

27 As well as noting it’s intuitive appeal (and widespread acceptance), Smith provides an argument for accepting this principle (2018, pp. 4–5). For discussion, see Kyburg (1970), Pollock (1983), Foley (1993), Douven and Williamson (2006).

28 It has also been suggested, by an anonymous reviewer, that because there are objections to iterative principles, like WW, this suggests that they are not highly intuitive. It is important to recognise, however, that arguments against a view (even highly compelling ones) need not displace our intuitions. There are compelling arguments against many of our strongest intuitions, including the intuition that there is an external world, as the sceptic argues, and the intuition at the very heart of this discussion: that some normative claims are true (Olson, 2014; Streumer, 2017). Typically, these arguments do not undermine our intuitions, however: even those who reject normative truth tend to acknowledge that it is an extraordinarily difficult pill to swallow. So, the fact that there are objections to WW does not show that it is not intuitive.

29 For further discussion on the distinction between internalism and externalism see Alston (1986), BonJour (1980) and (2010), Brown (2007), Conee and Feldman (2001), Goldberg (2007) Goldman (1979) and (1999) and Kornblith (2001).

30 Similarly, Raz (1994) and Nagel (2023) think that a necessary condition of moral truth (a subset of normative truth) is that they are intelligible to us and Williams (1979) is plausibly interpreted as arguing that, if P is a reason to φ then it must be possible to φ for P. Each of these views suggest that these normative truths must be within our epistemic reach.

31 Moreover, similar iterative principles have been argued for, from an externalist perspective (see Gibbons, 2006).

32 For this reason, it is also not solved by Jack and Khuramy (2025) who purport to solve Suikkanen’s regress by rejecting the claim that the states of information must be distinct.

33 Note that this is distinct from Wright’s suggestion that if P is warranted then we are warranted in believing that P is superassertible (1992, pp. 55–61). This, of course, does not mean that if P is warranted then P is superassertible. Rather, that if P is warranted then ‘P is superassertible’ is also warranted.

34 All of the same can be said of IW.
